# Diagnosis and Treatment of VOD/SOS After Allogeneic Hematopoietic Stem Cell Transplantation

**DOI:** 10.3389/fimmu.2020.00489

**Published:** 2020-04-03

**Authors:** Francesca Bonifazi, Francesco Barbato, Federico Ravaioli, Mariarosaria Sessa, Irene Defrancesco, Mario Arpinati, Michele Cavo, Antonio Colecchia

**Affiliations:** ^1^Institute of Hematology “L. and A. Seràgnoli”, S. Orsola-Malpighi University Hospital, Bologna, Italy; ^2^Department of Medical and Surgical Sciences, University of Bologna, Bologna, Italy; ^3^Institute of Hematology “L. and A. Seràgnoli”, Department of Experimental, Diagnostic and Specialty Medicine, Alma Mater Studiorum-Bologna University School of Medicine S. Orsola's University Hospital, Bologna, Italy; ^4^Department of Molecular Medicine, University of Pavia, Pavia, Italy; ^5^Gastroenterology Unit, Borgo Trento University Hospital, Verona, Italy

**Keywords:** VOD/SOS, HSCT, defibrotide, elastometry, liver stiffness measurement

## Abstract

Hepatic veno-occlusive disease (VOD) or sinusoidal obstruction syndrome (SOS) is a rare complication characterized by hepatomegaly, right-upper quadrant pain, jaundice, and ascites, occurring after high-dose chemotherapy, hematopoietic stem cell transplantation (HSCT) and, less commonly, other conditions. We review pathogenesis, clinical appearance and diagnostic criteria, risk factors, prophylaxis, and treatment of the VOD occurring post-HSCT. The injury of the sinusoidal endothelial cells with loss of wall integrity and sinusoidal obstruction is the basis of development of postsinusoidal portal hypertension responsible for clinical syndrome. Risk factors associated with the onset of VOD and diagnostic tools have been recently updated both in the pediatric and adult settings and here are reported. Treatment includes supportive care, intensive management, and specific drug therapy with defibrotide. Because of its severity, particularly in VOD with associated multiorgan disease, prophylaxis approaches are under investigation. During the last years, decreased mortality associated to VOD/SOS has been reported being it attributable to a better intensive and multidisciplinary approach.

## Introduction

Hepatic veno-occlusive disease (VOD), also known as sinusoidal obstruction syndrome (SOS), is a clinical syndrome occurring after high-dose chemotherapy, hematopoietic stem cell transplantation (HSCT) (1, 2), and, less commonly, after ingestion of toxic alkaloids (toxic injury) (3), after high doses of radiotherapy (4) or liver transplantation (5). Clinical diagnosis criteria include hepatomegaly, right-upper quadrant pain, ascites, and jaundice (6), although anicteric forms may occur, particularly, but not exclusively among pediatric population (7). The onset or the progression can be complicated by a multiorgan disease (MOD), characterized by functional disorders affecting lungs (pleural effusion, pulmonary infiltrates, hypoxia), kidneys (renal insufficiency/failure), and central nervous system (confusion, encephalopathy). Multiorgan disease is associated with high mortality rate (exceeding 80%), and it has been identified as the best predictive marker of severe VOD/SOS (8–10).

In HSCT patients, endothelial cell injury leads to loss of sinusoidal wall integrity, endothelial cell detachment, sinusoidal obstruction, and development of postsinusoidal portal hypertension (PH) (11). The incidence of posttransplant VOD/SOS is highly variable, ranging from 5.3% (12) to 13.7% (9) to higher percentages, according to transplant settings and different studies; particularly in pediatric high-risk populations, the incidence could be 20 to 30% up to 60% (7, 13–15).

Transplant outcome is significantly affected by VOD/SOS occurrence, where the mortality rates can reach up to 80% in the severe forms, in older series (9), whereas more recent studies report lower mortality rates (16, 17), in patients treated with defibrotide. Early diagnosis and treatment are positively correlated to a survival benefit (16). Treatment includes supportive care, intensive treatment, and specific drug therapy.

## Pathophysiology

**Figure 1:**
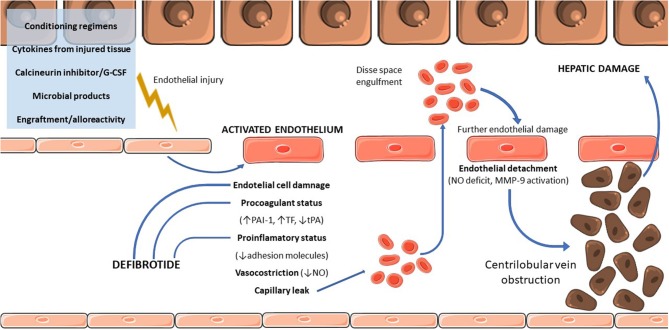
Physiopathology of VOD/SOS. G-CSF, granulocyte colony-stimulating factor; PAI, plasminogen activator inhibitor-1; TF, tissue factor; tPA, tissue plasminogen activator; MMP-9, matrix metallopeptidase 9.

The initial step of VOD/SOS pathogenesis is the injury of sinusoidal endothelium of the liver (Figure 1) leading to loss of endothelial cell cohesions: gaps appear in the endothelial barrier, and red blood cells pass through these gaps and accumulates in the Disse space, causing the detachment of the endothelial cells with downstream embolization of the centrilobular vein and subsequent postsinusoidal obstruction (18).

Several causes (Figure 1) are incriminated into initial endothelial damage, including conditioning regimens (19), cytokines produced by injured tissues (20), endogenous microbial products migrating through damaged mucosal barriers (21), drugs used during the transplant [such as granulocyte colony-stimulating factor (G-CSF) or calcineurin inhibitors] (22–24), and the engraftment process itself (25). Conditioning regimens have a crucial role in the pathogenesis as highlighted by the increased risk of VOD/SOS associated with higher plasma levels of cytotoxic drugs, such as busulfan or metabolites of cyclophosphamide (26). Chemotherapy drugs are metabolized by the cytochrome P450 complex, producing toxic metabolites subsequently converted to non-toxic ones by the glutathione (GSH) enzymatic system and then eliminated (27). Centrilobular regions of the liver are poor in GSH, making them more sensitive to toxic agents and explaining the predominant damage of centrilobular regions (28, 29). Moreover, a GSH S-transferase M1 null genotype reducing the detoxifying capacity of the liver parenchyma predisposes to SOS/VOD (30), and the reduced detoxifying ability due to immature enzymatic system could, at least partially, explain the higher incidence of VOD/SOS in children (13).

Some clinical observations led to the hypothesis that alloreactivity plays a role in VOD/SOS. Incidence of VOD/SOS is higher after allogenic compared to autologous HSCT and is higher in patients receiving a transplant from a mismatched unrelated donor (31). These observations are supported by findings in experimental models where endothelial cells are targets of alloreactive T cells (32).

Endothelial cells after HSCT show signs of injury characterized by procoagulant and proinflammatory status (Figure 1). This status is confirmed by the presence of increased levels of circulating markers of endothelial activation after HSCT, such as endothelial procoagulant factors and adhesion molecules (20), circulating endothelial cells (33), endothelial progenitor cells (34), and microparticles (35).

Endothelial cell detachment seems to be correlated with nitric oxide deficiency caused by postconditioning toxicity (36). Nitric oxide deficiency promotes increased endothelial cell production of matrix metalloproteinase 9 (MMP-9) that seems to be strongly involved in VOD/SOS development, probably promoting the endothelial cell detachment. The role of MMP-9 in the VOD/SOS pathogenesis is supported by the evidence that the *in vivo* inhibition of MMPs completely prevents its occurrence (37).

Along with the embolization by detached endothelial cells, blood flow obstruction is promoted by the proliferation of perisinusoidal stellate cells and subendothelial fibroblasts in the terminal hepatic vein followed by the deposition of the extracellular matrix (38). Then perivenular fibrosis spreads into the liver parenchyma (39). All these events lead to a block in liver blood outflow, with progressive obliteration of the venules and centrilobular sinusoidal, causing hepatic congestion and the development of postsinusoidal PH (40).

Because of the central role of endothelial injury in its pathogenesis, VOD/SOS is now classified as a transplant-related endothelial dysfunction, as well as posttransplant microangiopathy, idiopathic pneumonia, diffuse alveolar hemorrhage, and engraftment syndrome (11).

## Clinical Presentation and Diagnosis

The clinical presentation of VOD/SOS is the consequence of the PH, being characterized by rapid weight gain, tendentially unresponsive to diuretics, hyperbilirubinemia, painful hepatomegaly, and ascites. It generally occurs within 21 days after transplant, late-onset VOD/SOS is nowadays recognized as distinct VOD/SOS feature by recent diagnostic criteria elaborated by the European Society for Blood and Marrow Transplantation (EBMT) (41) (Table 1). It has been already reported that late VOD/SOS occurs at least in 39.3% and 16.7%, respectively, in the adult and pediatric setting (16).

**Table 1:** Modified seattle, Baltimore, and EBMT diagnostic criteria in adults **(A)** and in children **(B)**.

**(A) ADULTS**			
**Modified Seattle criteria**^**a**^	**Baltimore criteria**^**a**^	**EBMT criteria**^**a**^	
Presentation within 20 d from HSCT of ≥2 of the following: – Bilirubin >2 mg/dL – Hepatomegaly, right-upper quadrant pain – Weight gain >2% over baseline due to fluid retention	Within 21 d from HSCT bilirubin ≥2 mg/dL and at least 2 of the following: – Painful hepatomegaly – Weight gain >5% – Ascites	Classical VOD/SOS^a^ Within 21 d from HSCT bilirubin ≥2 mg/dL and ≥2 of the following: – Painful hepatomegaly – Weight gain >5% – Ascites	Late-onset VOD/SOS^a^ Classical SOS beyond day 21, OR Histologically proven SOS OR ≥2 of the classical criteria AND ultrasound (US) or hemodynamic evidence of SOS
**(B) CHILDREN**			
No time onset limitation for SOS/VOD occurrence			
The presence of ≥2 of the following paramethers^b^: • Unexplained refractoriness to platelets transfusions defined as ≥1 weight-adjusted platelet substitution/day to maintain institutional transfusion guidelines.^c^ • Otherwise unexplained weight gain on 3 consecutive days despite the use of diuretics or a weight gain >5% above baseline value • Hepatomegaly (best if confirmed by imaging such as US, CT or MRI) above baseline value measured pre-HSCT • Ascites (best if confirmed by imaging such as US, CT or MRI) above baseline value measured pre-HSCT • Increase of bilirubin above baseline value on 3 consecutive days or bilirubin ≥2 mg/dL within 72 h			

a *These symptoms/signs should not be attributable to other causes*.

b *With the exclusion of other potential differential diagnoses*.

c *One or more weight-adjusted platelet substitution/day to maintain institutional transfusion guidelines*.

*CT, computed tomography; HSCT, hematopoietic stem cell transplantation; MRI, magnetic resonance imaging; US, ultrasonography*.

The onset of VOD/SOS can be either smoldering or disruptive, ranging from mild forms spontaneously resolving within few weeks to severe forms with organ damage and MOD. Multiorgan disease, involving generally pulmonary and renal functions, can rapidly occur, significantly worsening the outcome (17, 41, 42). Because of the high mortality rate of severe VOD/SOS, daily monitoring for prompt detection of symptoms, such as jaundice, hepatomegaly, fluid overload with weight gain and ascites (42), is required. Although it remains a life-threatening condition, progresses in the management of severe VOD/SOS improved the outcome compared to the past (43).

The “traditional” diagnosis of VOD/SOS is based on fulfillment of either Baltimore (44) or modified Seattle criteria (6) (Table 1) and the exclusion of differential diagnosis.

Several conditions, such as fluid overload, constrictive pericarditis, ascites of different origin (pancreatic, chylous), drug-induced cholestasis and more generally drug-induced liver injury (DILI), cholangitis lenta, sepsis, infectious hepatitis, parenteral nutrition, cholestasis, and hepatic graft-versus-host disease (GvHD), can mimic VOD/SOS and still make real-life differential diagnosis a true challenge or pitfall.

The main difference between the two diagnostic systems is hyperbilirubinemia being mandatory in the Baltimore criteria, which implies longer time waiting for its development or intrinsically more aggressive forms. Up to 30% of children with VOD/SOS was anicteric (7, 45, 46) compared to 12% of adults. The clinical scenario can be variable, in particular in children where anicteric forms are not rare (13, 47) and dynamically changing.

For these reasons, the EBMT proposed, both in adult (Table 1A) and in pediatric (Table 1B) setting, new different diagnostic criteria and a scale for severity grading of suspected VOD/SOS (13, 41).

The EBMT criteria for adult patients (41) foresee two clinical entities: the classical VOD/SOS appearing within 21 days after HSCT with bilirubin ≥2 mg/mL and two of the following criteria: painful hepatomegaly, weight gain, and ascites. The late-onset VOD/SOS occurs beyond 21 days after transplantation and potentially presents as follows:

1. Same feature as the classical one,
2. It should be histologically proven, and
3. Two out of four criteria for the classical VOD/SOS (bilirubin ≥2 mg/mL, weight gain >5%, painful hepatomegaly, and ascites) plus hemodynamic or ultrasound (US) evidence of VOD/SOS.

In the pediatric setting (13), there are no distinctions related to the time of onset, and no time limitations are given. The fulfillment of at least two of the following criteria is required for diagnosis: the unexplained consumptive and transfusion-refractory thrombocytopenia, an otherwise unexplained weight gain on 3 consecutive days despite the use of diuretics or a weight gain 5% above the baseline value, hepatomegaly (best if confirmed by imaging) above the baseline value, ascites (best if confirmed by imaging) above the baseline value, and rising bilirubin from the baseline on 3 consecutive days or bilirubin ≥2 mg/dL within 72h.

The main differences between the diagnostic criteria of adult and children are the bilirubin increase, which can be missing mainly in the pediatric setting, in a significant proportion of cases and the presence of refractory thrombocytopenia. It should be reminded that the criteria have been established from different panels of experts, following a consensus-based approach; the refractoriness of thrombocytopenia to transfusion has been called in to discussion also for the adult criteria system but not finally adopted as a criterion because of lack of panel consensus. These criteria need to be further validated by forthcoming prospective studies (48).

Both adult and pediatric criteria have been associated to severity grading scales that are related to the dynamic changes, mainly the evolution of hepatic and renal function tests (Table 2). The speed of changes is considered a warning sign belonging to higher severity grading scale (for suspected VOD/SOS) and hence supporting early treatment initiation with potential clinical outcome improvement. This score system can be also used in case of suspected VOD/SOS, before patients meet the diagnostic criteria, especially before day 21 (41).

**Table 2:** EBMT criteria for severity grading of suspected VOD in adults **(A)** and in children **(B)**.

	**Mild**	**Moderate**	**Severe**		**Very severe**
**(A) ADULTS**					
Time since first symptoms	>7 d	5–7 d	≤ 4 d		Any time
Bilirubin (mg/dL)	≥2 to <3	≥3 to <5	≥5 to <8		≥8
Kinetics of bilirubin increase			Doubling in 48 h		
AST, ALT ( × UNV)	≤ 2	>2 to ≤ 5	>5 to ≤ 8		>8
Weight gain (%)	<5	≥5 to <10	≥5 to <10		≥10
Creatinine ( × baseline pre-HSCT)	<1.2	≥1.2 to <1.5	≥1.5 to <2		≥2 or other data of MOD
**(B) CHILDREN**					
Liver function tests (AST, ALT, GLDH)^a^	≤ 2 ×	>2 and ≤ 5 ×		>5 ×	
Persistent platlets refractoriness^a^	<3 d	3–7 d		>7 d	
Bilirubin (mg/dL)^a^^,^ ^b^	<2			>2	
Ascites^a^	Minimal	Moderate		Need of paracentesis	
Kinetics of bilirubin increase					Doubling within 48h
Coagulation	Normal		Impaired		Impaired coagulation with need of replacement of coagulation factors
Renal function GFR (mL/min)	89–60	59–30	29–15		<15
Pulmonary function (oxygen requirement)	<2 L/min	>2 L/min	Invasive pulmonary ventilation (including CPAP)		
CNS impairment		Absent			New onset cognitive impairment

*Patients belong to the category that fulfills ≥2 criteria. If patients fulfill ≥2 criteria in two different categories, they should be classified in the most severe category, in the presence of two or more risk factors for SOS, patients should be in the upper grade*.

a *Presence of two or more of these criteria qualifies for an upgrade to CTCAE level 4 (very severe SOS/VOD)*.

b Excluding preexistent hyperbilirubinemia due to primary disease.

*ALT, alanine transaminase; AST, aspartate transaminase; CNS, central nervous system; CPAP, continuous positive airway pressure; CTCAE, Common Terminology Criteria for Adverse Events; GFR, glomerular filtration rate; GLDH, glutamate dehydrogenase; MOD, multi-organ dysfunction*.

The EBMT diagnostic criteria for adults include a late-onset VOD/SOS where both histology and US attain key roles for the diagnosis itself. In pediatric setting, the role of imaging has been significantly upgraded, as suggested by the EBMT diagnostic criteria, which recommend hepatomegaly and ascites to be confirmed by imaging during the clinical course and immediately before HSCT (13).

Among imaging techniques, US is certainly one of the most commonly studied as it allows assessment of both parenchymal and vascular changes; it is cheap and can be used bedside. However, even though US has been recognized as an EBMT diagnostic criterion, its role is restricted to diagnosis confirmation, when clinical signs are already noticeable. Ultrasound and Doppler US can easily detect the typical signs of PH such as ascites, hepatomegaly, splenomegaly, and dilatation of portal vein, which are commonly present in symptomatic VOD/SOS. The first article describing systematically these typical US and US Doppler diagnostic criteria was published by Lassau et al. (49). The prospective study included 100 patients having undergone HSCT; 25 of 100 patients developed VOD/SOS. The authors used seven morphologic and seven Doppler criteria to define the value of US in the prediction, diagnosis, and prognostic assessment of VOD/SOS. Based on these 14 criteria, a diagnostic score was then produced; a score of 6 had a sensitivity of 83% and a specificity of 87%. However, at the best of US performance, ~20% of the VOD/SOS could be misdiagnosed according to the Lassau score. A recent article by Park et al. (50) confirmed that some morphological parameters such as ascites and gallbladder wall thickening were significantly associated (odds ratio, respectively, 56.3 and 36.3) to VOD/SOS diagnosis. Nishida et al. (51) proposed a novel scoring system (HokUS-10) based on 10 US variables, which was able to predict VOD/SOS diagnosis with sensitivity of 100% and specificity of 95.8% in 10 patients. Although HokUS-10 score is easier to apply than the Lassau score, it still has to be adequately validated. There is much evidence on the utility of US imaging as a diagnostic tool; nevertheless, its role is still controversial because of lack of reproducibility and the requirement need of an expert sonographer, especially for US Doppler. Furthermore, some US Doppler signs (e.g., patency of paraumbilical vein) appear when an advanced stage of VOD/SOS has already been developed; thus, its application may be very limited to early diagnose or to anticipate the clinical VOD/SOS diagnosis. The use of ultrasonographic contrast agent, which is able to assess the hepatic vascularization, has been used to facilitate the diagnosis and to evaluate treatment response (52, 53) in VOD/SOS setting.

Because magnetic resonance and computed tomography represent the gold standard techniques for focal liver lesions identification, particularly in cancer staging and surveillance, their use is still pivotal in post-HSCT VOD/SOS (42, 54, 55). However, the potential role of these imaging techniques can be further increased in all types of VOD/SOS (56). Major limitation for a broader use is related to logistic issues, mainly in critical patients.

Because of the potential complications of hepatic biopsy in thrombocytopenic patients (i.e., hemorrhage, hemobilia, shock), the possibility of a histologic diagnosis of VOD/SOS is quite limited to well-trained centers with dedicated multidisciplinary team and cannot be considered a routine practice. Transjugular biopsy can limit the risk of bleeding and allow the measurement of the hepatic venous pressure gradient (HVPG), although the risk of unreadable specimens can be accounted (57). Hepatic venous pressure gradient is the hallmark of PH: its measurement is a very specific tool for VOD/SOS diagnosis, and values >10 mm Hg predict VOD/SOS with good level of accuracy and specificity (58). The main limitation consists in being an invasive procedure.

In patients with advanced chronic hepatic disease, the measurement of PH via HVPG has been replaced by hepatic stiffness measurement performed by elastography, which is a non-invasive method. Liver stiffness measurement (LSM) by transient elastography has been introduced several years ago to stage liver diseases (59); since then, numerous experiences have demonstrated a good correlation between liver stiffness and liver disease grading (60). Thus, LSM progressively allowed reducing the number of liver biopsies performed in patients with advanced liver disease. Moreover, it was observed that LSM could also be useful to measure PH, because it closely correlates with HVPG (61). Elastography was used to predict VOD/SOS in HSCT patients. Recent studies (62–64) investigated the predictive role of LSM changes, assessed by transient elastography (TE) or shear wave elastography, in post-HSCT VD/SOS in pediatric and adult patients. Liver stiffness measurement values assessed by TE in healthy subjects without liver pathology range between 4.3 and 5.3 kPa (65, 66), whereas a threshold of 21 kPa holds a high specificity (>90%) and can be used to confirm the presence of clinically significant portal hypertension (67, 68). In HSCT patients, LSMs were carried out at baseline and once a week after HSCT. Only in patients who developed VOD/SOS, LSM values markedly increased compared to previous measurement (from 10.3–59.3 vs. 3.5–7.5 kPa) (62, 63). Liver stiffness measurement increases from 1 to 15 days before clinical VOD/SOS diagnosis and most intriguingly LSM decreased after the start of defibrotide treatment parallel to clinical signs of VOD/SOS (e.g., bilirubin, weight) (63–69). Based on these results, it was speculated that LSM, a non-invasive method, executable bedside, can be useful to perform both a preclinical diagnosis of VOD/SOS and to monitor treatment response. Main limitations for a wide application of this method are the need of a specific training of the operator, the presence of significant amount of ascites, and a body mass index >30 kgm^2^. Based on preliminary results, an Italian national multicenter prospective trial (“ElastoVOD/SOS Study,” ClinicalTrial.gov NCT03426358) is actually running, aimed to confirm the prognostic role of LSM in a prospective multicenter context.

Several biomarkers (70) have been proposed for VOD/SOS diagnosis and/or prevention; they are markers of hemostasis and coagulation such as plasminogen activator inhibitor 1 (PAI-1) or other markers of endothelial injury, such as elevated levels of von Willebrand factor, thrombomodulin, soluble intercellular adhesion molecule 1, suppressor of tumorigenicity 2, angiopoietin 2, hyaluronic acid (HA), or markers of inflammation [interleukin 6 (IL-6), IL-10, CD97].

The increased level of PAI-1 antigen is the most studied marker for its role as a predictor of VOD/SOS (71–74), whereas a decrease of its level has been correlated with better treatment outcome (75). Anyway, the proteomic-based approach published by Akil et al. (76) failed to include PAI-1 in the final predictive model. In this model only l-ficolin, HA, and vascular cell adhesion molecule 1 showed a prognostic value for diagnosis. Available data on single or combined panel of biomarkers for VOD/SOS are still inconclusive, and a wide application in the real world is so far marginal.

## Incidence and Risk Factors

The incidence of VOD/SOS after transplantation varies substantially from 2 to 60% (6, 7, 12, 13, 16, 47) because of both different setting of patients and transplant procedures and of application of different diagnostic criteria.

The incidence of VOD/SOS is higher in children than in adults (7, 9, 13–16, 47), although a retrospective analysis of a large Italian pediatric cohort (47) found a surprisingly very low incidence of VOD/SOS [2% (95% confidence interval, 1.7–2.5)].

Risk factors are generally classified as either patient related or transplantation related (77). Among the former ones, age, Karnofsky index, any preexisting liver disease, altered liver function tests, advanced hematological disease, second transplant thalassemia and ferritin level, and abdominal radiation are risk factors reported in literature since the last two decades.

The use of new immunotherapies for the therapy of acute leukemias, such as gemtuzumab ozogamicin for acute myeloid leukemia and inotuzumab ozogamicin for acute lymphoblastic leukemia, is associated with a significant increase of VOD/SOS risk (77–79), mainly related to the subsequent HSCT. In this respect, avoidance of more than two inotuzumab ozogamicin cycles and double alkylators in the preparing regimen and the use of ursodeoxycholic acid are recommended in patients suffering from relapsed/refractory acute lymphoblastic leukemia undergoing allogeneic HSCT after inotuzumab ozogamicin treatment (80).

The following transplantation-related risk factors should be mentioned (77): allogeneic vs. autologous transplant, mismatched/haploidentical transplant, T-replete transplants, and myeloablative-preparing regimen containing either busulfan or total body irradiation.

The odds ratios of each risk factor reported by the review from Dalle and Giralt (77) are those reported from each reference, *sic et simpliciter*, without a risk score–building purpose.

Recently, the Center for International Blood and Marrow Transplant Research developed a risk score built on a large population series of more than 13,000 patients (81). Younger age, positive hepatitis B/C serology, lower Karnofsky index, use of sirolimus, disease at transplant, and myeloablative-conditioning regimen were associated to higher risk of VOD/SOS. The authors did not include pretransplant therapies impacting on VOD/SOS, so the applicability of this model to patient receiving either gemtuzumab ozogamicin or inotuzumab ozogamicin is still unknown. Prospective validation of risk factors is yet to be completed and needs further assessment to provide a more precise estimation of the magnitude of each risk factor (70).

## Treatment and Outcome

The treatment of VOD/SOS includes supportive and intensive care in addition to the specific therapy with defibrotide.

Supportive care and clinical monitoring are primary issues in the management of VOD/SOS throughout the whole HSCT course, in order to promptly capture clinical diagnostic criteria, to timely record all dynamic changes and to follow both the response to treatment and disease progression. Daily reports of several parameters, such as abdominal circumference, weight, and diuresis, are recommended (13, 41). The nurse group of the Italian Society of Bone Marrow Transplantation elaborated an operational flowchart for a dynamic nursing monitoring of patients with suspected or proven VOD/SOS (82). Supportive care includes a careful evaluation of fluid balance with diuretics, as well as all therapeutic measures aimed to reduce the discomfort of massive ascites, pleural effusion, hypoxia, pain, and renal dysfunction such as paracentesis, thoracentesis, oxygen therapy according to the respiratory parameters, analgesic therapy, hemodialysis, or hemofiltration. A transfer to the intensive care unit can be required. The therapy at the intensive care unit is symptomatic and may differ among centers.

Defibrotide is the only registered drug for the treatment of moderate/severe VOD/SOS; it is a mixture of polydeoxyribonucleotide, mainly single-stranded, derived from the porcine intestinal mucosa. Its mechanism of action is not yet fully understood (83, 84). Oligonucleotides interact with heparin-binding proteins such as fibroblast growth factors, which exert fibrogenetic as well as angiogenetic effects with endothelial stabilization. Moreover, defibrotide acts as an antithrombotic and profibrinolytic drug; it reduces platelet adhesion and activation, without systemic anticoagulant effects, by means of inhibition of PAI-1, thrombin, and leukocyte adhesion process (via inhibition of P-selectin expression), and also decreases vascular permeability and apoptosis due to calcineurin inhibitors and chemotherapy, without interfering with antitumor effect of cytotoxic drugs (85). Because of the capacity of defibrotide to protect endothelium from toxic, inflammatory, and ischemic damage, its potential therapeutic use has been tested, some decades ago, in several vascular disorders such as thrombophlebitis (86, 87), in postsurgery deep vein thrombosis prophylaxis (88, 89), and peripheral arterial diseases (90) with significant benefits. It has been used, even in a pivotal way, in acute myocardial infarction (91), in postthrombolysis reocclusion of coronary (92), ischemic damage of the liver (93), diabetic microangiopathy, and Reynaud phenomenon (94).

The efficacy and safety of defibrotide in the setting of VOD/SOS, especially after HSCT, have been extensively evaluated by different authors. The first study is a historically controlled multicenter open-label phase III study (95) recruiting patients from 1995 to 2008; participating centers prospectively enrolled patients with established hepatic VOD/SOS to receive defibrotide 25 mg/kg per day, whereas the placebo cohort (32 patients) was retrospectively identified from 6,867 medical charts of HSCT patients by blinded independent reviewers in order to minimize the selection bias. The unusual study design (retrospective vs. prospective comparison) is due to the refusal of participating centers to accept a prospective randomization with placebo resulting unethical (orphan disease with high mortality). This study adopted the VOD/SOS diagnosis criteria, and severe VOD/SOS was defined as a VOD/SOS complicated by MOD. The primary endpoint was 100-day mortality; secondary endpoints were 100-day complete response (CR) rate and 6-month overall survival. The study demonstrated both 100-day survival and CR benefit favoring the defibrotide arm (38.2 vs. 25.0% and 25.5 vs. 12.5%, respectively). Median duration of therapy was 21.5 days, and 10.7% of patients discontinued defibrotide for treatment-related adverse event (AE). Adverse events were similar in the two arms, particularly hemorrhagic events (64% in the experimental arm vs. 75% in the historical control arm). Pulmonary alveolar hemorrhage occurred in 11.8 and 15.6% of the patients, gastrointestinal bleeding in 7.8 vs. 9.4%, and cerebral hemorrhage in 2.9 vs. 3.1%, respectively, in the experimental and control arms.

Concurrently the aforementioned phase III study, an international compassionate use program (CUP) (17), has been implemented, aimed to ensure drug supply to a wider range of transplant centers across the world. Transplant centers adhering to the CUP program enrolled patients developing severe VOD/SOS either after HSCT or after radiotherapy/chemotherapy. Both the Baltimore- and Seattle-modified (6, 44) diagnosis criteria were used; when the Seattle criteria were not met, the presence of US changes or histological diagnosis could be sufficient for patient recruitment and drug supply. Severe VOD/SOS was defined by the presence of MOD or by >30% of predicted risk retrospectively evaluated according to the Bearman model (96). Defibrotide doses ranged from 10 to 80 mg/kg, because no specific treatment protocol has been adopted. Participating centers voluntarily provided demographic and clinical data for the analysis. Overall 1,169 patients received at least one dose of defibrotide, whereas data were finally retrieved on 710 patients. Six hundred eighty-nine of 710 patients developed VOD/SOS after HSCT: 499 after an allogeneic HSCT, and 112 after autologous HSCT; 60% were transplanted for acute lymphoblastic leukemia, and 57% of the study population was adults (≥18 years old). Two hundred ninety-two of 710 patients were treated for a severe VOD/SOS. One hundred-day survival was 54% in the overall population (58% of those patients receiving defibrotide at the dose of 25 mg/kg) and was higher in the pediatric cohort (65.4 vs. 46.1%), in the group without MOD (64.7 vs. 39.7%), and in patients developing VOD/SOS after a non-HSCT therapy (74.2 vs. 67.5%). Adverse events occurred in 51% of patients, whereas overall discontinuation of the drug occurred in 28%; 9% of patients discontinued defibrotide because of AEs, mainly hemorrhages (gastrointestinal). No clinically meaningful trends in AE occurrence were identified by gender, age, or dose group.

The third study was a prospective open-label, single-arm study in an expanded access program (16) enrolling, from 2007 to 2016, patients with hepatic VOD/SOS, both post-HSCT and non-HSCT treatments, with the aim to evaluate 100-day overall survival (primary endpoint) and safety of defibrotide given at the dose of 25 mg/kg for at least 21 days. The inclusion criteria changed over time: initially, VOD/SOS should be diagnosed according to the Baltimore criteria by day +35 post-HSCT or by biopsy as well as MOD (by day +45 post-HSCT); then, VOD/SOS was diagnosed based on Seattle criteria, with onset after day +35, secondary to non-transplant treatment, also including VOD/SOS without MOD. A total number of 1,137 patients were enrolled, 1,000 with VOD/SOS after HSCT (85% allogeneic HSCT and 15% autologous HSCT). The pediatric group represented 82% of postautologous HSCT VOD/SOS and 52.3% of postallogeneic HSCT VOD/SOS. One hundred-day overall survival was 58.9% in the whole population, 68.5% in patients who developed VOD/SOS without MOD, and 49.5% in patients with MOD; VOD/SOS was significantly associated with MOD occurrence in all transplant types and all age groups. Late-onset VOD/SOS was more frequent in adults than in children (39.3% of adult patients and 16.7% of children) and was associated with lower survival only in the pediatric group. Earlier initiation of defibrotide treatment was significantly associated with higher day +100 survival (*P* < 0.001). Treatment-emergent AEs (in patients who received at least one dose of defibrotide) were more frequent in adults than in children (77.9 and 65.5%, respectively) and in patients with MOD (75.2% overall, 81% in adults, and 70.5% in children). Twenty-one percent of patients had at least one treatment-related AE (TRAE), which represented the reason for treatment discontinuation in 12.4% of patients. Treatment-related AEs were not different according in relation to VOD/SOS time of onset. The most important TRAEs were pulmonary hemorrhage (4.6%), gastrointestinal hemorrhage (3.0%), epistaxis (2.3%), and hypotension (2.0%).

A postmarketing phase IV study on defibrotide has been required by French regulatory authorities as a source of real-world data (48). Patients treated with defibrotide as prophylaxis were included, although there is no registration of defibrotide for this indication. In this study, VOD/SOS diagnosis was performed according to the EBMT criteria and the primary endpoints were both 100-day survival and 100-day complete response of severe VOD/SOS. Three hundred twenty-four French patients received defibrotide from July 2014 to October 2018; 40 developed severe VOD/SOS, and 120 after HSCT; overall, 105 patients developed a severe/very severe VOD/SOS (89 after HSCT). More than 30% of patients with VOD/SOS showed a concomitant MOD. One hundred-day survival in the overall population (140 patients), in severe VOD/SOS, and in very severe VOD/SOS were 58%, 79%, and 34%, respectively. The proportion of patients experiencing any AEs was 54% in the overall population. The study is still active, and definitive data are forthcoming.

Data from these important studies are quite superimposable and further confirmed by a systematic review of the literature, which found out 100-day survival of 41% in patients with MOD and 71% in those without MOD (97).

Corticosteroids, which have been used both in adult (98) and pediatric (99) setting, achieved the 2C level of recommendation in British guidelines (100); their use should be cautiously considered because of the increased risk of infections. Tissue plasminogen activator and N-acetylcysteine are not recommended for increased bleeding risk and lack of efficacy, respectively (100).

In case of no response and progression of VOD/SOS, the prognosis is dismal, and few further treatments are available, with limited efficacy. Transjugular intrahepatic portosystemic shunting placement has been reported in few anecdotal cases in literature for the treatment of VOD/SOS, but currently its use is not recommended because of poor outcomes (101). It has been considered sometimes when a severe VOD/SOS refractory to medical treatment occurred in a liver transplant recipient (102). Similarly, the role of orthotopic liver transplantation is controversial; its use has been described in few case reports in patients with severe VOD/SOS associated with life-threatening liver failure (103).

## Prophylaxis

Several pharmacological approaches have been tested with the purpose of preventing VOD/SOS, including heparin, antithrombin, prostaglandin E1, pentoxifylline, and ursodeoxycholic acid (9, 96). All these agents showed little or no efficacy or caused intolerable rates of adverse effects for a prophylactic strategy apart from ursodeoxycholic acid, which is recommended by British guidelines (100). Unfractionated heparin and low-molecular-weight heparin have been extensively studied, including some randomized trials, but with inconclusive results (12, 104–108). No efficacy was demonstrated for antithrombin and pentoxifylline (109–111). Also, the use of prostaglandin E1 was abandoned because of inconclusive results and excess of toxicity (112–114). The use of ursodeoxycholic acid (UDCA) in VOD/SOS prophylaxis has been investigated, in comparison to placebo, in three different randomized trials. Two of them (115, 116) demonstrated a significant reduction of VOD/SOS incidence in the UDCA arm; one revealed no differences between the two arms (117). A meta-analysis of the three trials comparing UDCA with placebo supported the use of UDCA as a possible effective prevention strategy, also because of its safety profile (118). Another randomized study compared prophylactic use of UDCA in association with heparin against heparin alone and revealed no differences in VOD/SOS incidence between the two groups (119).

The use of defibrotide as prophylactic agent has been tested in several retrospective studies (120–122) and in one prospective randomized trial in the pediatric setting. This phase III, randomized, open-label, multicenter trial compared defibrotide to placebo as VOD/SOS prophylaxis in pediatric patients undergoing allogeneic or autologous HSCT (7). In this study, each patient had one or more VOD/SOS risk factor including preexisting hepatic disease, second myeloablative transplant, allogeneic transplant for leukemia beyond second relapse, conditioning with busulfan and melphalan, prior treatment with gemtuzumab ozogamicin or a diagnosis of primary hemophagocytic lymph histiocytosis, adrenoleukodystrophy, or osteopetrosis. The trial included 365 patients younger than 18 years equally allocated in two arms. Patients in the treatment group received defibrotide (DF) 25 mg/kg per day in four divided intravenous infusions, starting with the initiation of conditioning regimen and continuing for 30 days after transplantation or, if discharged from hospital before 30 days after HSCT, for at least 14 days. The primary endpoint was the incidence of VOD by 30 days after HSCT. Twenty-two patients (12%) in the DF group developed VOD/SOS compared with 35 patients (20%) in the control group (*Z*-test for competing risk analysis *P* = 0.0488; log-rank test *P* = 0.0507).

Based on these results, the British Committee for Standards in Hematology and the British Society for Blood and Marrow Transplantation guidelines recommend the use of defibrotide for VOD/SOS prophylaxis in children undergoing HSCT with at least one risk factor for VOD/SOS (evidence IA) (100).

In adults, evidences are far less conclusive, and consistent results from randomized trials are still lacking. Even if some retrospective studies suggest a possible role of defibrotide for prophylaxis of VOD/SOS, particularly in high-risk patients, there is no clear evidence of its efficacy (123–125). There is no physiological reason why defibrotide should not work for VOD/SOS prophylaxis in adult, but it is yet to be proved if a prophylactic strategy would grant a better outcome than an early treatment strategy. A prospective randomized trial is ongoing, aimed to clarify these issues (ClinicalTrials.gov NCT02851407).

## Conclusions

Diagnosis of VOD/SOS is currently based mainly on clinical criteria; biomarkers for VOD/SOS diagnosis are not yet validated. The most reliable imaging method supporting VOD/SOS diagnosis is US, which is now recognized as an essential diagnosis criterion of late-onset VOD/SOS and highly recommended to assess hepatomegaly and ascites in children. Nevertheless, VOD/SOS diagnosis remains difficult in real-life setting, despite the availability of different diagnostic criteria systems; differential diagnosis is quite challenging because several other conditions could meet the VOD/SOS criteria, such as sepsis, cholangitis lenta, constrictive pericarditis, hepatic GvHD, hepatitis, or DILI. Moreover, more than one complication can occur simultaneously in the same patient leading to a substantial delay of final diagnosis. When clinical criteria are not fully met, invasive diagnostic methods are still hard to be widely used because they need well-trained multidisciplinary teams to perform and read biopsy or HVPG measurement. Even in the presence of these facilities, however, patients with suspicious or proven VOD/SOS can be critically instable, and the risk of further severe procedure-related complications cannot be prevented.

For these reasons additional tools for the diagnosis are most welcome. Elastometry is a non-invasive method to perform LSM, which is a validated surrogate of HVPG, in advanced chronic liver disease. If ongoing studies confirm the role of elastometry in HSCT patients, this non-invasive imaging technique will allow an earlier VOD/SOS diagnosis and an accurate monitoring of treatment response. The use of elastometry underpinned the importance of a multidisciplinary approach to VOD/SOS with specialists in radiology, hepatology, intensive care, and nephrology supporting and helping physicians performing HSCT in the management of VOD/SOS. Anyway, the use of elastometry for VOD/SOS diagnosis and for treatment response evaluation deserves further validation by prospective studies.

In the past, the mortality risk for patients who develop posttransplant VOD/SOS with MOD, typically characterized by pulmonary and/or renal dysfunction, has been estimated to be >80% (8–10). In more recent reports, mortality rates are significantly lower: 22 and 35% at 100 days and 5 years, respectively, in the retrospective large Italian pediatric cohort (47), and 49.5% estimated survival at 100 days in the T-IND study (16).

Decreased mortality can be attributed to a better intensive care, to increasing proportion of centers with multidisciplinary teams, to a wider use of risk stratification, to earlier treatment. Prevention of MOD occurrence and progression of severity grading significantly increased survival in all HSCT transplants settings (16, 49). Finally, a greater knowledge on risk factors will lead to a more tailored approach to both prevention and treatment of VOD/SOS.

## Author Contributions

FBon and AC conceived and designed the study. FBon wrote the first draft. AC, MS, FBar, ID, FR, and MC wrote sections of the manuscript. All the authors contributed to manuscript revision, read and approved the submitted version.

### Conflict of Interest

FBon and AC participated on advisory boards and received speaker fess from JAZZ Pharmaceuticals. The remaining authors declare that the research was conducted in the absence of any commercial or financial relationships that could be construed as a potential conflict of interest
